# Geographical structure of endosymbiotic bacteria hosted by *Bathymodiolus* mussels at eastern Pacific hydrothermal vents

**DOI:** 10.1186/s12862-017-0966-3

**Published:** 2017-05-30

**Authors:** Phuong-Thao Ho, Eunji Park, Soon Gyu Hong, Eun-Hye Kim, Kangchon Kim, Sook-Jin Jang, Robert C. Vrijenhoek, Yong-Jin Won

**Affiliations:** 1 Interdisciplinary Program of EcoCreative, The Graduate School, Ewha Womans University, Seoul, 03760 Korea; 2 Division of EcoScience, Ewha Womans University, Seoul, 03760 Korea; 3 Division of Polar Life Sciences, Korea Polar Research Institute, 26 Songdomirae-ro, Yeonsu-gu, Incheon, 21990 Republic of Korea; 4 Monterey Bay Aquarium Research Institute, Moss Landing, CA 95039 USA

**Keywords:** Chemosynthetic symbiosis, Deep-sea hydrothermal vent, *Bathymodiolus* mussels, Sulfur-oxidizing endosymbiont, Gammaproteobacteria, Geographical population structure

## Abstract

**Background:**

Chemolithoautotrophic primary production sustains dense invertebrate communities at deep-sea hydrothermal vents and hydrocarbon seeps. Symbiotic bacteria that oxidize dissolved sulfur, methane, and hydrogen gases nourish bathymodiolin mussels that thrive in these environments worldwide. The mussel symbionts are newly acquired in each generation via infection by free-living forms. This study examined geographical subdivision of the thiotrophic endosymbionts hosted by *Bathymodiolus* mussels living along the eastern Pacific hydrothermal vents. High-throughput sequencing data of *16S* ribosomal RNA encoding gene and fragments of six protein-coding genes of symbionts were examined in the samples collected from nine vent localities at the East Pacific Rise, Galápagos Rift, and Pacific-Antarctic Ridge.

**Results:**

Both of the parapatric sister-species, *B. thermophilus* and *B. antarcticus*, hosted the same numerically dominant phylotype of thiotrophic Gammaproteobacteria. However, sequences from six protein-coding genes revealed highly divergent symbiont lineages living north and south of the Easter Microplate and hosted by these two *Bathymodiolus* mussel species. High heterogeneity of symbiont haplotypes among host individuals sampled from the same location suggested that stochasticity associated with initial infections was amplified as symbionts proliferated within the host individuals. The mussel species presently contact one another and hybridize along the Easter Microplate, but the northern and southern symbionts appear to be completely isolated. Vicariance associated with orogeny of the Easter Microplate region, 2.5–5.3 million years ago, may have initiated isolation of the symbiont and host populations. Estimates of synonymous substitution rates for the protein-coding bacterial genes examined in this study were 0.77–1.62%/nucleotide/million years.

**Conclusions:**

Our present study reports the most comprehensive population genetic analyses of the chemosynthetic endosymbiotic bacteria based on high-throughput genetic data and extensive geographical sampling to date, and demonstrates the role of the geographical features, the Easter Microplate and geographical distance, in the intraspecific divergence of this bacterial species along the mid-ocean ridge axes in the eastern Pacific. Altogether, our results provide insights into extrinsic and intrinsic factors affecting the dispersal and evolution of chemosynthetic symbiotic partners in the hydrothermal vents along the eastern Pacific Ocean.

**Electronic supplementary material:**

The online version of this article (doi:10.1186/s12862-017-0966-3) contains supplementary material, which is available to authorized users.

## Background

Symbiosis involving chemoautolithotrophic bacteria plays a predominant role supporting the diverse invertebrate fauna that flourishes at deep-sea hydrothermal vents, hydrocarbon seeps, whale and wood falls, and organically enriched shallow water sediments [1]. The invertebrate animals use three different means to acquire the symbionts. For example, vent clams in the family Vesicomyidae, transmit their bacteria vertically through an ovarial pathway [2–4], though some species might acquire bacteria laterally from unrelated hosts or from the environment, a process known as leaky vertical transmission [5]. Most chemosymbiotic taxa, such as the iconic vent tubeworm *Riftia pachyptila*, acquire their symbionts horizontally via infections by a free-living stage of microbe [6]. Various transmission modes pose different trade-offs (reviewed in [7]). Vertical transmission provides ‘symbiont assurance’ resulting in the joint dispersal of hosts and symbionts, but it might constrain host species to a narrow ecological niche. Horizontal acquisition risks the failure of dispersing propagules acquiring the ‘right kind’ of bacteria when they settle in new habitats, but it creates opportunities to adopt diverse locally adapted strains of symbiotic bacteria wherever they settle [8, 9]. Because leaky vertical transmission involves vertical and horizontal components, it allows hosts to replace their symbionts with locally optimal strains, but it also engenders a risk of infections by “cheater” strains and pathogens [7]. Although numerous studies have examined the population genetics and geographical connectivity of various invertebrate hosts (reviewed in [10]), only a few studies have examined their bacterial partners [11–16]. Often the genetic markers employed to study the symbionts were limited in number and too conservative to adequately discern fine-scale geographical structure.

Herein, we attempt to assess geographical structure and dispersal of horizontally transmitted chemosynthetic bacteria hosted by parapatric sister-species of *Bathymodiolus* mussels. Various members of the mytilid subfamily Bathymodiolinae that commonly dominate vent and seep communities worldwide host a diverse suite of chemoautotrophic eubacteria capable of oxidizing H_2_S, CH_4_ or H_2_ gases [17–24]. Apparently, their exceptional capacity for adopting phylogenetically diverse and locally adapted strains of bacteria has contributed to their rapid global radiation during the Middle Eocene and Early Oligocene Epochs [9, 25].

A combination of microscopy and molecular evidence indicate that bathymodiolin larvae acquire their endosymbionts from the environments in which they settle [8, 9, 26–29]. Initial infections of larvae occur in a range of epithelial tissues and then shift to the developing gills, where the bacteria proliferate [30–32]. Researchers hypothesized that dispersing mussel larvae might carry hitchiking symbionts from natal sites to new habitats in which they settle [8], but the genetic evidence was insufficient and inconclusive. Regardless, environmental symbiont acquisition renders bathymodiolins susceptible to metabolic cheaters and pathogens [7], but successful infections appear to be highly specific to potential symbiont species [31]. Unlike the experimentally tractable legume/rhizobium system [33–35], signalling pathways and metabolic interactions that control specificity have not been investigated in the mussel/symbiont mutualism.

**Fig. 1:**
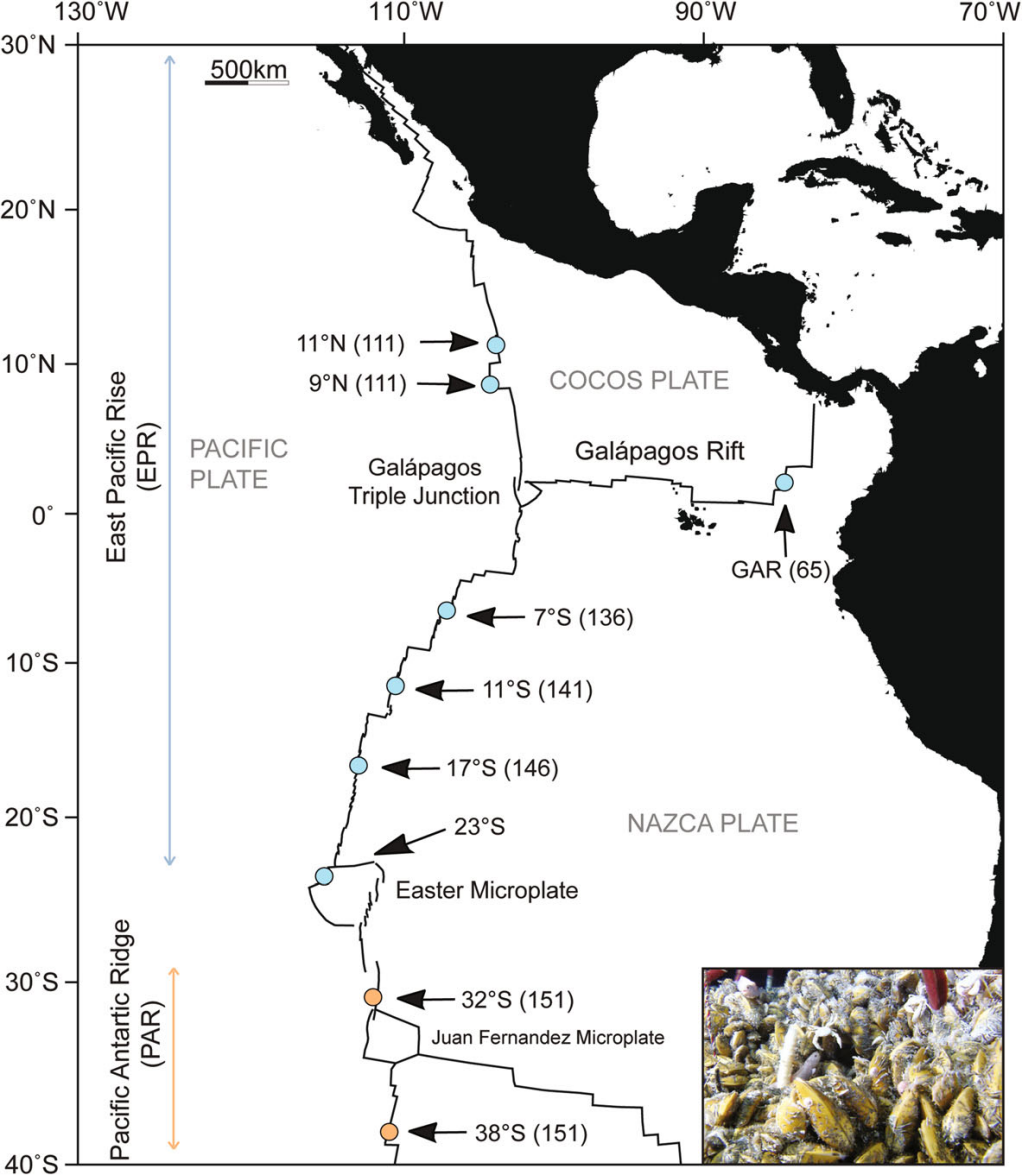
*Bathymodiolus* sample localities along the East Pacific Rise (EPR), Galápagos Rift (GAR), and Pacific-Antarctic Ridge (PAR). Numbers in parenthesis indicate tectonic spreading rates in mm/year. Lines perpendicular to ridge axes indicate fracture zones. Inset illustrates a *B. thermophilus* bed at 9°N latitude (Photo credit: Stephen Low Productions, Courtesy of R. A. Lutz, Rutgers University)

The goal of this study was to determine whether geographic barriers known to act on subdivision of the *Bathymodiolus* host species also govern genetic structure of the thiotrophic endosymbionts. Closely related sister-species of mussels are abundant at southeastern Pacific vents (Fig. 1). *Bathymodiolus thermophilus* Kenk & Wilson 1985, occupies the Galápagos Rift (GAR, at 0° latitude) and East Pacific Rise (EPR, between 13°N latitude and 18°S), whereas *Bathymodiolus antarcticus* Johnson, Won, Harvey, and Vrijenhoek 2013, occupies a northeastern extension of Pacific-Antarctic Ridge (PAR, between 32and 38°S latitude). The host species contact one another and hybridize at 23°S along the northwestern margin of the Easter Microplate [36]. Strong cross-axis currents intersect the ridge axes in this uplifted region, creating dispersal barriers for a number of vent-restricted animals (reviewed in [10]). However, a gap exists in our knowledge about the symbiotic bacteria hosted by these *Bathymodiolus* sister-species, particularly with respect to their host specificity and biogeographic population structure. Although the northern species, *B. thermophilus*, is known to harbor a single thiotrophic symbiont species [37], symbionts associated with the southern species, *B. antarcticus* are poorly understood. To ascertain whether the southern mussel is infected by the same symbiont species, and if physical barriers to dispersal similarly impede the northern and southern symbiont populations, we used high-throughput DNA sequencing of the *16S* rRNA encoding gene and six protein-coding genes isolated from symbiotic bacteria sampled throughout the known ranges of their mussel hosts. We applied anlaysis of molecular variance (AMOVA) methods to estimate the proportions of symbiont genetic diversity: (1) contained between the northern and southern regions; (2) contained within population samples within regions; and (3) housed within individual host mussels.

## Methods

### Sampling

Oceanographic expeditions conducted between 1990 and 2005 sampled *Bathymodiolus* mussels from hydrothermal vent fields distributed along the Galápagos Rift (GAR), the East Pacific Rise (EPR), and the Pacific-Antarctic Ridge (PAR) (Fig. 1). Details regarding geographical coordinates, bathymetric depths, and sampling methods were previously reported [36]. Following recovery of the research submarine HOV *Alvin*, mussels were immediately transferred to buckets containing 2 °C filtered seawater. Gill tissue samples were dissected from adult mussels and preserved directly in cryovials containing 95% ethanol or frozen at −80 °C. The DNeasy Blood and Tissue Kit (QIAGEN Inc., Valencia, CA) were used to extract total DNA from approximately 25 mg of gill tissue from each specimen.

### Community analysis of bacteria from *Bathymodiolus* gill tissues

A subsample of 45 mussels from seven vent fields was screened for eubacteria-specific *16S* rRNA sequences (Table 1A). We designed new PCR primers (with barcodes and linkers) to span the 27F and 516R region of *16S* rRNA encoding gene conventionally used for eubacterial ribotyping (Additional file 1: Table S1, barcodes in Additional file 1: Table S2A). Amplicons from 45 independent reactions were pooled and sequenced using 454 GS-FLX titanium sequencing machine (Roche, Branford, CT). We used PyroTrimmer [38] to remove the barcode, linker and primer sequences, and trim the 3′-ends of sequences with low-quality reads (i.e. average score < 20 nucleotides per 5-bp sliding window). Ambiguous sequences and those shorter than 300 bp were discarded. The de novo chimera detection algorithm incorporated in UCHIME program [39] was used to detect and discard chimeric reads. To detect different phylotypes, sequence reads were trimmed to have 300 bp and identical or different only by insertion/deletion of bases were grouped together. The most frequent representative sequence of *16S* rRNA encoding gene was deposited in GenBank [GenBank: KX987235] and the other rest variant sequences have been deposited in the Dryad data repository as a polymorphic table. To see if the size selection (300 bp) effects on the final result, the similar trimming and same downstream analyses were also applied to the original 454-pyrosequencing data with different criteria for DNA lengths (250, 350, 400, and 450 bp). The predominant sequence in each phylotype group was used to represent the group in subsequent phylogenetic analyses. Taxonomic affiliation of each representative sequence was determined by RDP classifier [40] against *16S* rRNA training set 16 of RDP project. We initially analyzed the forward and reverse sequences, but ultimately used the reverse sequences alone as they provided improved recognition of phylotypes.

**Table 1:** Sampling information and high throughout data of (A) *16S* ribosomal RNA genes and (B) protein-coding genes of *Bathymodiolus* mussel’s thiotrophic endosymbionts, and (C) *Bathymodiolus* samples examined in this study

(A)					
Vent	Dive log^a^	Number of host individuals		Count of reads	
9°N	A2498, A3540	12		14,260	
7°S	A3320	3		2178	
11°S	A3323	8		8104	
17°S	A3327	7		7024	
23°S	A4096	2		2027	
32°S	A3339, A3340	9		6649	
38°S	A4091	4		3179	
(B)					
Vent	Dive log	MID sequences^b^	Number of host individuals	Count of reads	
11°N	A2226	ACGTAGATCGT	8	93,943	
9°N	A3540	ACTACGTCTCT	8	105,970	
GAR	A2223	ACGCGTCTAGT	8	82,618	
7°S	A3320	ACTATACGAGT	8	73,839	
11°S	A3323	ACTCGCGTCGT	8	75,903	
17°S	A3327	ACACGTAGTAT	8	99,215	
23°S	A4096	ACACTACTCGT	8	46,566	
32°S	A3340	ACGACACGTAT	8	117,794	
38°S	A4091	ACGAGTAGACT	8	92,461	
(C)					
Vent locality	Latitude	Longitude	Depth (m)	Dive log	Date
Galápagos Rift (GAR)	0°47.9′N	86°09.2′W	2486	A2223	May 28, 1990
East Pacific Rise (EPR)					
11°N	11°24.9′N	103°47.3′W	2515	A2226	May 4, 1990
9°N	9°50.5′N	104°17.5′W	2525	A2498	Mar 6, 1992
	9°49.4′N	104°17.7′W	2518	A3540	Apr 14, 2000
7°S	7°25.0′S	107°48.6′W	2747	A3320	Dec 23, 1998
11°S	11°18.2′S	110°31.8′W	2669	A3323	Dec 27, 1998
17°S	17°24.9′S	113°12.2′W	2578	A3327	Dec 31, 1998
23°S	23°32.9′S	115°34.2′W	2595	A4096	Apr 1, 2005
Pacific-Antarctic Ridge (PAR)					
32°S	31°09.4′S	111°55.9′W	2332	A3339	Jan 15, 1999
	31°51.8′S	112°02.8′W	2331	A3340	Jan 16, 1999
38°S	37°40.4′S	110°52.6′W	2236	A4091	Mar 25, 2005

^a^Human-occupied deep-sea vehicle (HOV) *Alvin* dive numbers

^b^Multiplex Identifier sequences used for labeling of different populations for pooled 454-pyrosequencing

### Symbiont protein-coding genes

To further characterize the thiotrophic endosymbionts, we examined six protein-coding genes from 72 mussels sampled from nine vent fields, which included the subset of individuals examined for *16S* rRNA sequences (Table 1B). For each individual, the *16S* rRNA-coding and six protein-coding DNA sequences were amplified from a whole genomic DNA extract obtained from a single gill tissue sample. Symbiont-specific PCR primers and barcodes were designed to span at least 400 bp of each gene (Additional file 1: Table S1, barcodes in Additional file 1: Table S2B). These six genes were chosen from our unpublished genomic sequence (~2.7 Mb) of the thiotrophic endosymbionts (Won Y-J, unpublished data assembled by PacBio sequencing method from a host gill tissue collected at the latitude of 9°N, East Pacific Rise) under the following conditions: (1) existence as a single copy; (2) even representation of genomic positions (Additional file 2: Figure S1); and (3) diverse coverage of the functional category of bacteria such as transcription (*rpoD*), chaperones (*COI* and *dnaK*), carbohydrate transport and metabolism (*pgi* and *pykF*), and sulfur metabolism (*soxA*). Nested PCRs were conducted with two sets of primers for each gene. Primers used for the second PCR attached unique barcodes for eight individuals from each sample location (Additional file 1: Table S2B) to 5′-ends of the forward and reverse sequences.

We used a nested PCR protocol to preferentially amplify sequences known to mark *Bathymodiolus* thiotrophic symbionts and minimize potential contamination by sequences from environmental bacteria. The nested thermal cycling was performed in 20 μl of reaction with initial denaturation for 1 min at 94 °C; denaturation for 40 s at 92 °C, 60 s at 58 °C (35 cycles), extension for 1 min at 72 °C; and final extension for 7 min at 72 °C. All PCR products were then pooled according to sampling locations (nine vent localities as shown in Fig. 1). The next step involved ligation of MID adapters containing the recognition sequence for nine sampling sites (Table 1B). Ligation was performed according to the GS FLX short-gun DNA library preparation quick guide (Roche, Branford, CT). Once MID adapters were ligated, all amplicons were pooled into a single tube and sequenced using 454 GS-FLX titanium sequencing machine (Roche, Branford, CT).

We used UCHIME [39] to remove erroneous chimeric reads from the protein-coding sequences. A two-step algorithm was developed to correct sequencing errors (Additional file 2: Figure S2). First, we imported raw 454-pyrosequencing reads into Geneious v.6.1.5, and aligned them with the MUSCLE algorithm [41]. The barcodes and primers were removed and the remaining sequences were scanned for stop-codons or frame-shift errors. Second, we developed a Python script to correct probable sequencing errors (Additional file 3). Although the pyrosequencing error rate was unknown, a range of conservative error-correction criteria (1–10%) was applied to the data. For example, a 1% criterion treated nucleotide substitutions occurring at 1% within a population sample as sequencing errors and corrected them to the most frequent base at that position. The process was repeated for the 2–10% criteria and the effects of these criteria on estimates of *F*-statistics were compared (Additional file 2: Figure S3). Generally, pairwise *F*_*ST*_ estimates and hierarchical AMOVAs were robust to these criteria; so, we only report results from the 1% criterion.

Comparisons with partial genomic data from *Bathymodiolus* thiotrophs (Won Y-J, unpublished data), revealed that all of the 454-pyrosequencing reads of *pgi* exhibited complete deletion of a single ‘T’ in a poly ‘T’ track that would lead to premature termination the polypeptide. To restore the normal reading-frame we manually inserting a ‘T’ at the deletion site in all the *pgi* sequences. We also compared our *COI* and *dnaK* pyrosequences with Sanger-sequencing data from *B. thermophilus* thiotrophs [42] to confirm that our primer sets were specific for the bacteria genes (Additional file 2: Figure S4). Unfortunately, the remaining protein-coding genes could not be evaluated in this way due to an absence of published sequence data. The most frequent representative sequences of the six protein-coding genes were deposited in GenBank [GenBank: KX987236-KX987241], and the other rest variant sequences of each gene have been deposited in the Dryad data repository as polymorphic tables.

### Population genetics

We used Splitstree v.4.12 [43] to generate unrooted sequence networks for the six protein-coding genes. Genetic diversity indices, pairwise *F*_ST_ estimates, and the analysis of molecular variance (AMOVA) were obtained with Arlequin v. 3.5 [44]. We used the NUVEL-1A model option implemented in a web-based application (http://www.ldeo.columbia.edu/~menke/plates 2.html) to estimate seafloor spreading rates. Correlations between average sequence diversity (*H*) and seafloor-spreading rates were estimated with SPSS v.21 (IBM, Armonk, NY). Pairwise geographical distances between sample localities were estimated in Google Earth Pro v.7.1.2. Correlations between pairwise *F*_ST_ estimates and geographical distances were examined. Because hierarchical subdivision can also generate patterns resembling IBD signals [45], we used the stratified Mantel test implemented in GenoDive [46] to remove the effects of subdivision.

We used a vicariant event to estimate synonymous and nonsynonymous substitution rates for the protein-coding genes. The sequences were sorted into groups located north and south of the Easter Microplate, a geomorphological feature that is estimated to be 2.5–5.3 million years (MY) old [47, 48]. Assuming that no symbiont exchanges crossed this geographic barrier following the initial separation, we used the Nei and Gojobori [49] method to estimate corresponding substitution rates for each gene. A Python script developed for use with high-throughput genetic data can be provided upon request from Y-J Won.

## Results

### Community analysis of bacteria from *Bathymodiolus* gill tissues

The *16S* sequences revealed a single dominant eubacterial species occupying gill tissues of mussels sampled throughout the known ranges of the two hosts (Additional file 4: Table S3). After quality-control measures were applied to delete questionable sequences, we were left with 43,421 sequence reads of ≥300 bp length. Gammaproteobacteria overwhelmingly predominated, constituting 93.4 to 100% of the sequence reads from 45 mussels. Most of these sequences matched previously published phylotypes for thiotrophic endosymbionts hosted by *Bathymodiolus* mussels. Minor eubacterial classes found in these mussels included Betaproteobacteria (0–5.9%), Epsilonproteobacteria (0–4.1%), and Flavobacteriia (0–1%) (Additional file 4: Table S3). Further discussion of these minor eubacterial classes is premature, however. Their potential roles as symbionts, environmental contaminants, or pathogens, must be verified with fluorescence in situ hybridization (FISH) methods to determine their location internally or externally on gill tissues [21, 22]. Those analyses are beyond the scope of this population genetic study.

### Small subunit rRNA diversity in *Bathymodiolus* symbionts

Altogether, 94% of the 43,421 *16S* sequence reads could be grouped into seven relatively frequent phylotypes that differed by only one nucleotide to each other (Additional file 1: Table S4). Phylotypes HT1–HT7 were nearly identical to previously published Sanger sequences from endosymbionts hosted by EPR *B. thermophilus* (DQ321716) and PAR *B. antarcticus* (DQ321717) [27]. Phylotype HT1, which dominated all seven samples and constituted 93% of the total sequence reads (Additional file 1: Table S4), was 100% identical with a 300-bp segment from the published sequences. Sequence diversity of the seven-most frequent phylotypes was extremely low (mean *π*_population_ = 0.0001, and mean *H*_population_ = 0.043, Table 2 and Additional file 5: Table S5). Differentiation among the sampled populations was very small (mean pairwise *F*_ST_ = 0.048 ± 0.008 SD; Table 3) with one exception. Elevated pairwise *F*_ST_’s involving the small 7°S sample (mean *F*_ST_ = 0.106 ± 0.011 SD; *n = 3*) probably resulted from sampling error.

**Table 2:** DNA sequence diversity of *Bathymodiolus* endosymbionts

Locus		Sample localities								
		11°N	9°N	GAR^a^	7°S	11°S	17°S	23°S	32°S	38°S
*16S* rRNA encoding gene (300 bp)	*N* ^*b*^	-	13,310	-	2008	7531	6688	1899	6400	3046
	*h* ^*c*^	-	7	-	3	6	6	3	4	5
	*H* ^*d*^	-	0.030	-	0.157	0.020	0.029	0.026	0.009	0.029
	*π* ^*e*^	-	1.0E-04	-	5.3E-04	6.6E-05	9.5E-05	8.7E-05	3.0E-05	9.8E-05
*COI* Cytochrome C oxidase subunit I (443 bp)	*N*	3294	3692	1583	2246	2157	2545	1029	2332	737
	*h*	36	24	40	8	11	59	17	33	19
	*H*	0.80	0.70	0.72	0.77	0.68	0.86	0.84	0.67	0.73
	*π*	0.003	0.002	0.003	0.003	0.002	0.003	0.004	0.002	0.003
*dnaK* Chaperone protein (469 bp)	*N*	2465	3100	2995	2362	2671	1876	1967	2367	2300
	*h*	5	39	23	22	19	45	4	33	43
	*H*	0.42	0.78	0.62	0.80	0.67	0.65	0.45	0.82	0.83
	*π*	0.001	0.003	0.002	0.003	0.002	0.002	0.001	0.003	0.003
*pgi* Glucose-6-phosphate isomerase (441 bp)	*N*	1966	2986	2466	1972	1918	2668	1339	3747	1528
	*h*	35	30	57	16	16	70	11	151	103
	*H*	0.82	0.85	0.85	0.67	0.70	0.87	0.46	0.91	0.91
	*π*	0.004	0.004	0.004	0.003	0.003	0.004	0.002	0.008	0.006
*pykF* Pyruvate kinase (404 bp)	*N*	2234	3051	3475	2770	3020	2629	1602	3150	1934
	*h*	39	37	57	32	65	43	21	87	39
	*H*	0.75	0.81	0.77	0.74	0.90	0.79	0.79	0.88	0.78
	*π*	0.003	0.006	0.005	0.006	0.006	0.003	0.004	0.006	0.003
*rpoD* RNA polymerase, sigma D factor (388 bp)	*N*	3326	4328	3901	3650	4090	3717	2403	4797	2199
	*h*	30	26	25	26	22	42	27	18	42
	*H*	0.84	0.76	0.81	0.80	0.80	0.64	0.70	0.65	0.68
	*π*	0.004	0.003	0.004	0.004	0.004	0.002	0.004	0.002	0.003
*soxA* Sulfur oxidation protein A (361 bp)	*N*	4196	5036	4085	3763	4652	3370	2715	3879	4105
	*h*	6	9	12	7	3	5	8	4	6
	*H*	0.45	0.70	0.70	0.47	<0.01	0.34	0.03	0.03	0.17
	*π*	0.001	0.001	0.002	0.001	<0.001	0.001	<0.001	<0.001	<0.001

^a^Galápagos Rift

^b^Number of sequence reads

^c^Number of distinct DNA sequences estimated from the data with 1% of error-correction

^d^Diversity of sequences estimated from the data with 1% of error-correction

^e^Nucleotide diversity estimated from the data with 1% of error-correction

**Table 3:** Geographical distances (GEO) and genetic differentiation (*F*_ST_) between population samples. First listed matrix is below the diagonal and second matrix is above

Matrix		11°N	9°N	GAR	7°S	11°S	17°S	23°S	32°S	38°S
*16S*/GEO	11°N		208	1404	2035	2578	3341	4010	4772	5507
	9°N	-		1243	2035	2578	3197	3815	4564	5261
	GAR	-	-		4773	2337	3039	3660	4240	4787
	7°S	-	0.110	-		2387	1232	1947	2691	3379
	11°S	-	<0.001	-	0.114		744	1455	2233	2944
	17°S	-	<0.001^*^	-	0.094	<0.001^*^		701	1553	2272
	23°S	-	0.001^*^	-	0.061	0.002^*^	<0.001^*^		957	1662
	32°S	-	*0.003*	-	*0.132*	*0.001*	*0.003*	*0.005*		722
	38°S	-	*<0.001* ^*^	-	*0.069*	*0.002*	<*0.001* ^*^	*0.001* ^*^	0.005	
*COI*/*dnaK*	11°N		0.04	0.25	0.28	0.27	0.36	0.46	***0.92***	***0.92***
	9°N	0.03		0.10	0.15	0.15	0.20	0.25	***0.89***	***0.89***
	GAR	0.02	0.02		0.06	0.06	0.08	0.11	***0.91***	***0.91***
	7°S	0.31	0.36	0.34		0.07	0.07	0.14	***0.89***	***0.89***
	11°S	0.03	0.06	0.08	0.34		0.04	0.09	***0.90***	***0.90***
	17°S	0.10	0.12	0.11	0.12	0.12		0.03	***0.90***	***0.90***
	23°S	0.15	0.24	0.19	0.2	0.16	0.1		***0.92***	***0.92***
	32°S	***0.88***	***0.90***	***0.90***	***0.89***	***0.91***	***0.88***	***0.88***		0.15
	38°S	***0.87***	***0.90***	***0.88***	***0.88***	***0.90***	***0.86***	***0.85***	0.08	
*pgi*/*pykF*	11°N		0.17	0.12	0.15	0.10	0.19	0.12	***0.69***	***0.78***
	9°N	0.09		0.10	0.14	0.06	0.20	0.17	***0.64***	***0.70***
	GAR	0.16	0.04		0.09	0.08	0.09	0.08	***0.65***	***0.72***
	7°S	0.24	0.13	0.09		0.08	0.05	0.04	***0.58***	***0.66***
	11°S	0.25	0.11	0.08	0.02		0.14	0.09	***0.61***	***0.67***
	17°S	0.15	0.10	0.09	0.04	0.04		0.02	***0.63***	***0.73***
	23°S	0.23	0.15	0.13	0.07	0.05	0.06		***0.61***	***0.72***
	32°S	***0.78***	***0.78***	***0.77***	***0.78***	***0.78***	***0.78***	***0.78***		0.13
	38°S	***0.83***	***0.82***	***0.81***	***0.84***	***0.85***	***0.82***	***0.85***	0.05	
*rpoD*/*soxA*	11°N		0.27	0.30	0.26	0.23	0.21	0.18	***0.92***	***0.91***
	9°N	0.12		0.08	0.08	0.31	0.28	0.26	***0.88***	***0.87***
	GAR	0.10	0.14		0.04	0.39	0.33	0.33	***0.87***	***0.87***
	7°S	0.25	0.22	0.26		0.31	0.26	0.25	***0.91***	***0.89***
	11°S	0.09	0.07	0.09	0.18		0.24	0.01	***1.00***	***0.98***
	17°S	0.31	0.21	0.20	0.27	0.12		0.17	***0.96***	***0.94***
	23°S	0.24	0.15	0.25	0.24	0.11	0.15		***0.99***	***0.97***
	32°S	***0.81***	***0.82***	***0.83***	***0.81***	***0.81***	***0.85***	***0.81***		0.07
	38°S	***0.76***	***0.78***	***0.78***	***0.76***	***0.77***	***0.82***	***0.74***	0.04	

Note: Geographic distances in km

All *F*_ST_ estimates of protein-coding genes are significant after Bonferroni correction, but some (^*^) of the *16S* encoding gene are not significant after Bonferroni correction. Italicized *F*_ST_ represent comparisons between populations belonging to two geographical groups, EPR + GAR and PAR. Bold italicized *F*_ST_ estimates indicate highly genetic differentiation of functional genes between two populations, 23and 32°S, that are isolated from each other with the Easter Microplate intervening

Hierarchical AMOVA (Table 4A) revealed that none of the bacterial ribotype diversity resided in differences between host samples from the EPR versus PAR regions and only 3.13% resided in differences among samples within the regions. Remarkably, the remaining 98.05% of the ribotype variation was contained in the differences among symbionts within (89.52%) and among host individuals (9.8%) from vent localities (Table 4B).

**Table 4:** AMOVAs of *Bathymodiolus* mussels’ endosymbionts

Source of variation	Marker	d.f.	Sum of squares	Variance components	Percentage of variation
(A)					
Among groups (i.e. host species)	*16S* rRNA	1	0.38	0.00	−1.17
	*COI*	1	22,674.87	4.34	86.06
	*dnaK*	1	41,820.56	5.65	90.27
	*pgi*	1	38,679.04	4.89	81.25
	*pykF*	1	15,028.33	1.84	62.47
	*rpoD*	1	25,836.11	2.31	75.03
	*soxA*	1	25,088.60	2.00	85.89
Among populations within group	*16S* rRNA	5	13.01	0.00	3.13
	*COI*	7	1627.89	0.11	2.15
	*dnaK*	7	1552.40	0.09	1.44
	*pgi*	7	1532.95	0.10	1.67
	*pykF*	7	2287.06	0.12	4.22
	*rpoD*	7	3272.85	0.13	4.30
	*soxA*	7	2290.84	0.08	3.56
Among symbiont strains within population	*16S* rRNA	40,875	626.60	0.02	98.05
	*COI*	19,606	11,646.94	0.59	11.79
	*dnaK*	22,094	11,474.33	0.52	8.30
	*pgi*	20,581	21,164.98	1.03	17.08
	*pykF*	23,856	23,350.91	0.98	33.31
	*rpoD*	32,402	20,595.15	0.64	20.67
	*soxA*	35,792	8778.76	0.25	10.56
(B)					
Among populations (i.e. vent fields)	*16S* rRNA	6	12.14	0.00	0.68
	*COI*	8	1135.98	0.03	7.33
	*dnaK*	8	1461.87	0.04	10.10
	*pgi*	8	988.51	0.03	6.21
	*pykF*	8	1229.89	0.02	4.52
	*rpoD*	8	2157.00	0.04	9.16
	*soxA*	8	4994.22	0.13	36.15
Among host individuals within population	*16S* rRNA	38	52.80	0.00	9.80
	*COI*	61	2965.76	0.18	40.76
	*dnaK*	60	2852.45	0.16	37.43
	*pgi*	63	2242.95	0.13	27.48
	*pykF*	63	3398.70	0.18	38.11
	*rpoD*	62	4544.92	0.17	37.75
	*soxA*	61	3022.24	0.11	30.12
Among symbiont strains within host	*16S* rRNA	40,847	571.01	0.01	89.52
	*COI*	19,545	4572.94	0.23	51.91
	*dnaK*	22,034	4936.40	0.22	52.46
	*pgi*	20,746	6558.35	0.32	66.31
	*pykF*	23,793	6400.20	0.27	57.37
	*rpoD*	32,340	7867.61	0.24	53.09
	*soxA*	35,731	4392.82	0.12	33.73

### Diversity of protein-coding genes in *Bathymodiolus* symbionts

**Fig. 2:**
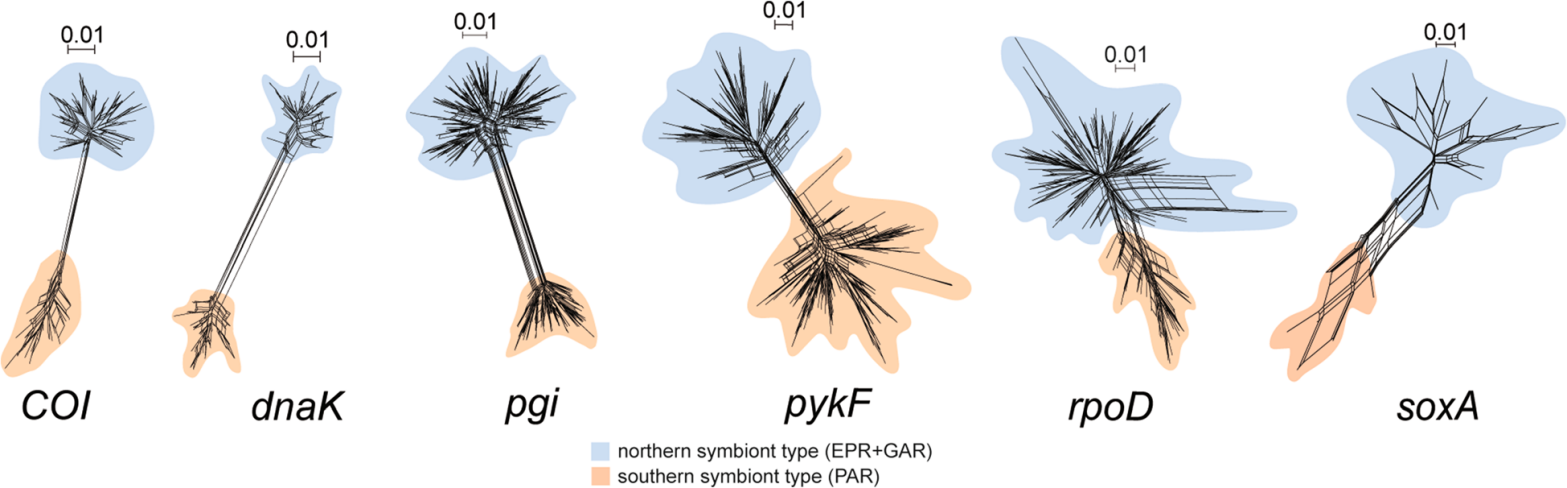
Sequence networks of six protein-coding genes of *Bathymodiolus* bacterial symbionts. Two representative geographical regions are overlaid on the networks with two different colors corresponding to the regional sampling sites as in Fig. 1: blue for northern symbiont type (EPR + GAR) and orange for southern symbiont type (PAR) harbored by *B. thermophilus* and *B. antarcticus* host mussels, respectively

Unlike the *16S* rRNA encoding gene, DNA sequence networks for six protein-coding gene segments (Fig. 2) all exhibited bifurcating patterns (northern and southern symbiont types in Fig. 1) corresponding with the northern (*n* = 56; EPR + GAR) and southern host species (*n* = 16; PAR). All but one of the nucleotide substitutions between the northern and southern symbiont lineages constituted synonymous substitutions (Additional file 1: Table S6). The *pgi* fragment alone showed a single fixed nonsynonymous substitution (Thr ↔ Ala). All other substitutions (fixed or polymorphic) in the six protein-coding genes were synonymous. Both *pgi* and *pykF* exhibited the greatest number of distinct sequences (*h*) (Table 2 and Additional file 6: Table S7). Except for *soxA*, haplotypic diversities (*H*) were relatively homogeneous across the sample locations. *SoxA* diversity was lowest at three southern locations. Pairwise *F*_ST_ estimates of the six protein-coding genes were much greater between the host groups (mean *F*_ST_ = 0.83 ± 0.095 SD) than among populations within groups (mean *F*_ST_ = 0.15 ± 0.042 SD) (Table 3).

Hierarchical AMOVA revealed that most of the diversity in protein-coding genes (61.97–90.65%) resided in differences between endosymbiont populations hosted by two mussel species (Table 4A). Only 1.43–4.34% resided among population samples within the host groups, and 7.92–34.07% resided among sequence reads within population samples. We further partitioned the within-sample latter component into within- and among-host individual components (Table 4B). An unexpectedly large proportion of variation resided in symbiont compositions among host individuals (27.48–40.76%), and the balance resided within hosts (33.73–66.31%). Frequencies of the most abundant protein-coding sequences varied greatly among host individuals across all nine of the population samples (Additional file 6: Table S7), resulting in the unexpectedly large among-host individual variability.

### Symbiont isolation-by-distance

We used a stratified Mantel procedure to test for an Isolation-by-Distance (IBD) pattern in the protein-coding genes. Because geographic subdivision can generate spurious IBD patterns [45], samples were partitioned into regions based on distributions of the mussel hosts. The averaged pairwise *F*_ST_ estimates within the partitions still presented significant positive correlations with distance (*r* = 0.439; *P* = 0.021). Consequently, an Isolation-by-Distance (IBD) pattern holds for *Bathymodiolus* symbiont populations.

### Mussels and symbionts in the hybrid zone

**Fig. 3:**
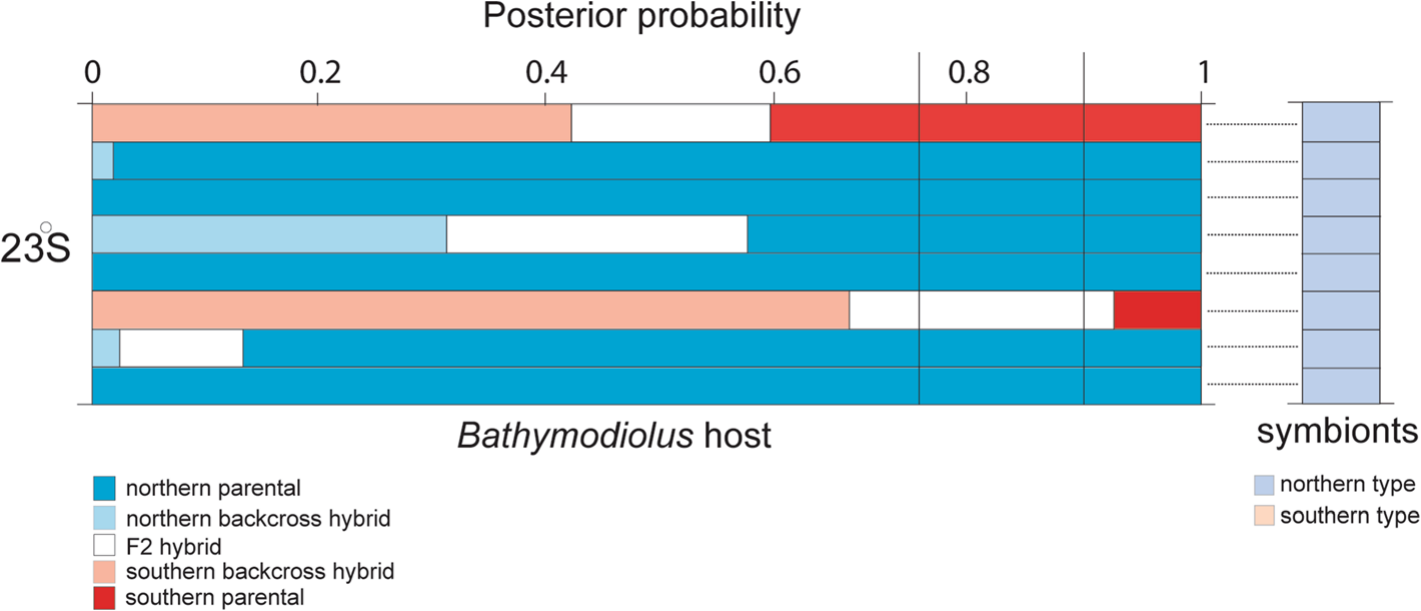
Genetic relationship between host mussels and their thiotrophic endosymbionts from the hybrid zone at 23°S. The left panel represents genetic assignments of eight individual mussels to five categories: *blue* = northern parental (*B. thermophilus*); red = southern parental (*B. antarcticus*), *black* = F1 hybrid; *white* = F2 hybrid, *light blue* = northern backcross hybrid, and pink = southern backcross hybrid [36]. *Vertical lines* mark 0.75 and 0.90 values of posterior probabilities for the assignments. The right panel represents the corresponding symbiont type of each host mussel. *Dark blue* color-codes for northern symbiont type (EPR) and orange color codes for southern symbiont type (PAR)

The hybrid zone population at 23°S latitude includes individuals with varying proportions of *B. thermophilus* and *B. antarcticus* genes [36]. To examine possible correspondence between the northern and southern symbiont strains and their respective hosts, we used the results of Johnson et al.’s [36] NEWHYBRIDS analysis that assigned individual mussels to putative parental, F1, F2, or backcross categories. The symbiont sequences we obtained from a random subsample of eight hybrid zone mussels only revealed the presence of the northern symbiont strain, regardless of the hosts’ genotypes (Fig. 3). It should be noted that two of the mussels had relatively high proportions of southern (*B. antarcticus*) genes, but they harbored the northern symbiont strain. The symbiont DNA sequences from this sample of eight mussels provided no evidence that the southern symbiont strain occurred in the 23°S mussels, but we cannot exclude the possibility that they might exist in the ambient environment.

### Nucleotide substitution rates

We estimated nucleotide substitution rates for the six protein-coding genes based on a vicariance associated with orogeny of the Easter Microplate, about 2.5–5.3 million years ago (MYA) (Table 5). Based on this range of dates, mean synonymous substitution rates were estimated as 0.77–1.62% per nucleotide per million years (MY). Likewise, nonsynonymous rates were estimated as 0.01–0.023% per nucleotide per MY, roughly 70 times slower than synonymous substitutions.

**Table 5:** Genetic distances and substitution rates of protein-coding genes of *Bathymodiolus* symbiotic bacteria between the two geographical groups, EPR + GAR and PAR

Gene	All codon positions	3rd pos.	1st and 2nd pos.	Synonymous distance (±SE) (syn. Substitution rate)	Nonsynonymous distance (±SE) (nonsyn. Substitution rate)
*COI*	0.0226^a^	0.0676	0.0001	0.102 (±1.37 × 10^–6^) (0.96 ^b^ − 2.041^c^ %)	7.31 × 10–5 (±6.47 × 10^–8^) (6.89 × 10^–4^–1.46 × 10^–3^%)
*dnaK*	0.0274	0.0751	0.0036	0.116 (±1.11 × 10^–6^) (1.10–2.33%)	2.94 × 10^–4^ (±1.18 × 10^–7^) (2.75 × 10^–3^–5.48 × 10^–3^%)
*pgi*	0.0261	0.0705	0.0038	0.105 (±1.62 × 10^–6^) (0.99–2.10%)	3.24 × 10^–3^ (±9.96 × 10^–8^) (3.06 × 10^–2^–6.48 × 10^–2^%)
*pykF*	0.0147	0.0.043	0.0005	0.046 (±2.03 × 10^–6^) (0.43–0.92%)	2.20 × 10^–3^ (±1.85 × 10^–7^) (2.08 × 10^–2^–4.40 × 10^–2^%)
*rpoD*	0.0149	0.0448	0.0000	0.067 (±9.65 × 10^–7^) (0.63–1.33%)	6.33 × 10^–5^ (±5.10 × 10^–8^) (5.98 × 10^–4^– 1.27 × 10^–3^%)
*soxA*	0.0124	0.0356	0.0008	0.051 (±3.43 × 10^–7^) (0.48–1.01%)	1.02 × 10^–3^ (±1.40 × 10^–7^) (9.65 × 10^–3^–2.04 × 10^–2^%)
Average	0.0197	0.0232	0.0015	0.081 (±1.24 × 10^–6^) (0.77–1.62%)	1.15 × 10^–3^ (±1.10 × 10^–7^) (0.01–0.02%)

^a^Nucleotide difference per site

^b^Substitution rate (percentage per nucleotide per million years) calibrated by the geological time of the formation of Easter Microplate, estimated as about 5.3 million years ago

^c^Substitution rate calibrated by 2.5 million years as an age of the Easter Microplate

## Discussion

The realization that *B. thermophilus* hosts a single thiotrophic symbiont species was identified by reverse transcription sequencing of small subunit rRNA with oligonucleotide primers [37]. Subsequent molecular studies confirmed this result [27], but the PCR/direct sequencing methods used in these studies have limited power to detect rare strains or species. The present high-throughput metagenomics analysis revealed that *B. antarcticus* and *B. thermophilus* both host a single overwhelmingly predominant ‘ribospecies’ of thiotrophic Gammaproteobacteria. We loosely use term ‘ribospecies’ [50, 51] to denote a grouping of *16S* rRNA phylotypes that share ≥97% sequence similarity.

Nonetheless, extensive sequence variation in six protein-coding genes revealed that this symbiont ‘ribospecies’ comprised two highly divergent evolutionary lineages that were geographically separated by the Easter Microplate, corresponding with parapatric distributions of the *B. thermophilus* and *B. antarcticus* hosts. On average, 80% of the protein-coding sequence diversity was contained in differences between these symbiont lineages and 17.2% resided in the differences within vent fields. Only 2.9% of the total diversity occurred among vent fields within geographical regions, but this small variance component manifested an Isolation-by-Distance (IBD) signal that was significant even after hierarchical subdivision was taken into account. Remarkably, an IBD pattern was not found in the *Bathymodiolus* hosts [36, 52]. Therefore, the bacteria appeared to exhibit more limited “realized” dispersal than the mussel hosts, which produce relatively long-lived planktotrophic larvae [53]. Johnson et al. [36] reported that *B. antarcticus* and *B. thermophilus* mussels contact one another and hybridize at 23°S, but we found no evidence that corresponding symbiont lineages were mixed at this locality. They also reported evidence for asymmetrical introgression of southern *B. antarcticus* alleles into northern *B. thermophilus*, but the northern and southern symbionts appear to be completely isolated. Our examination of host and symbiont genotypes in a sample of eight mussels from the hybrid zone provided no evidence for a host/symbiont specificity. Instead, we only found the northern symbiont lineage, despite the existence of some mussels with a large proportion of *B. antarcticus* genes (Fig. 3). The apparent absence of southern symbionts at this locality suggests the possibility of complete geographical isolation of the symbiont stains across the Easter Microplate region. Although, we do not know how these bacteria disperse, the present evidence does not support a hypothesis that the symbionts might be transported with dispersing mussel larvae [8].

The Easter Microplate boundary acts as a variable dispersal filter for a number of vent-restricted taxa. It separates sister-species pairs of bythograeid crabs [54, 55], and lepetodrilid limpets [56]. The boundary also separates genetically differentiated metapopulation segments of the siboglinid tubeworm *Tevnia jerichonana*, and the alvinellid palmworm *Alvinella pompejana*, but the degrees of differentiation do not warrant species recognition [57, 58]. In contrast, it is not associated with differentiation in *Branchipolynoe symmytilida*, a polynoid annelid that resides in the mantle cavities of mussels [59], or *Riftia pachyptila*, the giant siboglinid tubeworm that is emblematic of vents [60]. The isolating potential of this boundary is taxon-specific, reflecting complex interactions between the unique life histories of species, their historical distributions throughout southeast Pacific, and metapopulation processes related to regional extinctions, recolonization events, range expansions, and dispersal modes (reviewed in [10]).

Little is known, however, about the life history of these mussel symbionts. A free-living stage has been identified in vent habitats [42], and the early stages of infection was identified in juvenile mussels [31, 32, 61]. However, it is unknown if the symbiotic stages recycle to the free-living demographic component, as occurs in a siboglinid tubeworm symbiont [62]. Although the symbionts and mussel hosts might experience independent demographic processes, they appear to have experienced similar biogeographical histories. Orogeny of Easter Microplate region probably played a common role in vicariance of their northern and southern populations. This small tectonic plate is estimated to have formed 2.5 to 5.3 million years ago [47, 48] (Fig. 1). Its east and west rifts are connected by northern and southern transform faults [63]. Topographically elevated seamount chains extending east and west of the Microplate are believed to interrupt deep-ocean circulation, creating strong cross-ridge axis currents in the Easter Microplate region [64]. Empirical evidence based on the oceanic distribution of unique vent gases (i.e. Helium-3) supports the ocean circulation models [65]. Consequently, Won et al. [66] hypothesized that the strong cross-axis currents in this region create a contemporary barrier to dispersal for many vent-restricted animals, and particularly for species like mussels that produce planktotrophic larvae. Furthermore, the southern EPR and PAR exhibit superfast tectonic spreading rate of 141–151 mm/yr [67] that are believed to control the rate of habitat turnover in these regions [10, 68]. Regional differences in tectonic and volcanic activities could alter local geochemical conditions that, in turn, might affect the reduced allelic diversity of *soxA* in the SEPR and PAR endosymbionts (Additional file 6: Table S7). Further research on vent geochemistry in this region might shed some light on factors affecting the physiological ecology of these symbionts and their mussel hosts.

Assuming a 2.5–5.3 million-year (MY) time to the most recent common ancestor of the northern and southern symbiont lineages, we estimated synonymous substitution rates for the six protein-coding genes. The estimated range of rates, 0.77–1.62% per site per MY (Table 5), is comparable to estimates for synonymous substitution in other bacteria: 0.45% for genomic and 0.6–0.8% for protein-coding in *E. coli* [69, 70]; 0.82% for genomic and 0.39–0.8% for elongation factor Tu (*tuf*) in *Buchnera* [69, 71]. The estimated nonsynonymous substitution rates for *Bathymodiolus* endosymbionts (0.01–0.023% per site per MY, Table 5) were almost identical to rates for the *tuf* gene in *Buchnera* (0.013–0.025%, [71]).

Divergence of the northern and southern symbiont lineages was characterized by numerous fixed and polymorphic substitutions at synonymous sites. Only one fixed nonsynonymous substitution was found among the six nuclear gene fragments (Additional file 1: Table S6). The data provided insufficient statistical power to test for adaptive differentiation between the northern and southern symbiont alleles at these loci. Nonetheless, an absence of evidence for natural selection acting on these alleles does not preclude adaptive divergence between the northern and southern symbionts. The lineages might be differentially adapted to EPR and PAR environments or genetically co-adapted with their *B. thermophilus* and *B. antarcticus* hosts. Bathymetric variation probably does not contribute to divergence, as all the sampled sites fell within a narrow depth range of 2236–2747 m (Table 1C), but detailed comparative information about geochemical conditions in the two regions is lacking (e.g., [72–74]). Differentiation in the communities of Gamma- and Epsilon-proteobacteria hosted by western Pacific vent gastropods of the genus *Alviniconcha* to be triggered by local patchiness and regional scale differences in vent geochemistry [14]. Yet, *Alviniconcha* snails differ greatly from *B. thermophilus* and *B. antarcticus* mussels, which host a single predominant ‘ribospecies’ of Gammaproteobacteria. Perhaps the excess of synonymous substitutions observed in the present sample of genes from this bacterium only represents neutral differentiation between historically isolated populations. Although several physiological studies have been conducted for these uncultivable symbionts [75–77], comparative studies of temporal and spatial variation in vent biogeochemistry have not been undertaken. Examining a larger sample of protein-coding loci in these mussels or a very large number of genome-wide single nucleotide polymorphisms (SNPs) might provide the statistical power needed to conduct tests for adaptive differentiation (e.g., [78, 79]).

**Fig. 4:**
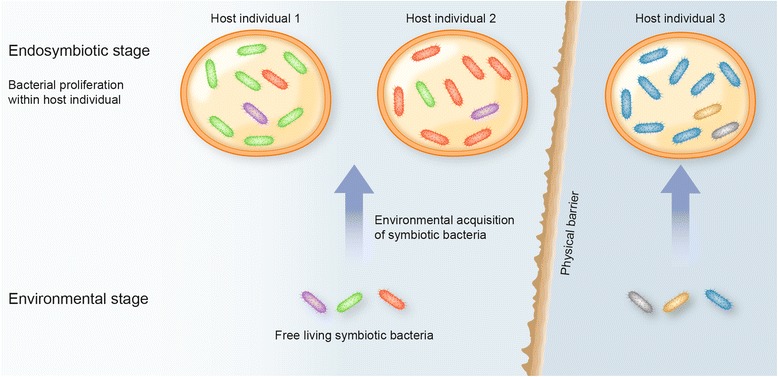
Model for the hierarchical differentiation of *Bathymodiolus* symbionts. The differently colored symbiotic bacteria represent different strains of the conspecific bacteria in both free-living and endosymbiotic phases. The large arrows represent environmental acquisition of free-living symbionts by the mussel hosts. An intervening physical barrier, in this case the Easter Microplate region, isolates EPR + GAR and PAR populations of the symbiotic bacteria

As previously noted, *Bathymodiolus* mussels acquire thiotrophic endosymbionts via infection by free-living stages that occur in the local environment. High levels of differentiation among host individuals within vent samples (35.28% of total variation; Table 4B) versus low differentiation among vent samples within a region (2.89% of total variation; Table 4A) appears to be anomalous, unless infections are a stochastic consequence of small-scale temporal and spatial genetic heterogeneity of the free-living bacterial strains (Fig. 4). Poisson sampling of the free-living bacterial population seems likely if they are relatively scarce. Fontanez and Cavanaugh [42] reported densities of ~1.8 × 10^6^ free-living bacteria with the appropriate ribotypes living in biofilms on basaltic blocks experimentally deployed at western Pacific hydrothermal vents. Ambient seawater sampled near adult mussels contained lower densities (~1.74 × 10^5^/l) that declined with distances from the *Bathymodiolus* patch. Once settling mussel larvae are infected, rapid proliferation of the bacteria would greatly enrich the abundance of a small number founding strains (e.g., [31, 32]). *Bathymodiolus* adults from the Mid-Atlantic Ridge were estimated to host ~2.5 × 10^12^ symbionts per individual [61]; thus, the high variance among host individuals probably reflects initial sampling bias associated with infections and secondary biases that accrue during enrichment. A random sample of host individuals from a given locality averages these individual sampling biases and provides a better estimate of strain frequencies at that locality. As long as the mussels were not sampled from a single potentially aberrant patch, these averages would explain the low among-locality variation along a ridge axis.

The adage, “Everything is everywhere, but the environment selects” ([80], p. 15), provides a useful and potentially falsifiable starting hypothesis for studies of microbial diversity [81]. Are the pre-infectious thiotroph strains identified in this study distributed evenly throughout a vent field, or does small-scale environmental heterogeneity in geochemical conditions favor different strains? The genetic composition of pre-infectious stages remains unknown, but small-scale heterogeneity in the chemistry of *Bathymodiolus* habitats, diffuse-flow low-temperature vents, does influence the distribution of free-living bacteria [82]. Habitat patchiness might contribute to the symbiont variance among host individuals in most of the samples. Conversely, the low among-host variance observed at 17and 38°S might have resulted from sampling of mussels from a single patch (Additional file 6: Table S7). Multiple sample chambers have been used with ROVs to characterize the small-scale patchiness in the symbionts hosted by siboglinid tubeworms and *Alviniconcha* snails [14, 15]. Unfortunately, they were not available on HOV *Alvin* during our 1999 through 2005 expeditions. Consequently, the present study represents a starting point for addressing these difficult questions. More directed efforts must be made to isolate biological subsamples and obtain corresponding biogeochemical data from discrete environmental patches, a goal for future studies.

## Conclusions

The previous understanding of extrinsic and intrinsic factors affecting the dispersal and evolution of chemosynthetic symbiotic partners has been mostly limited to invertebrate hosts due to the difficulties in sampling and culturing of the bacteria. Here, we attempted to overcome these methodological challenges through the combination of parallel DNA pyrosequencing, highly variable genetic markers, and appropriate geographical sampling of horizontally transmitted thiotrophic endosymbiotic bacteria of deep-sea hydrothermal vent invertebrate hosts, *Bathymodiolus* mussels (Mollusca: Mytilidae), in the eastern Pacific Ocean. The community analysis based on sequences of slowly evolving *16S* rRNA encoding gene confirmed that all the host individuals belonging to two allopatric host species, northern *B. thermophilus* and southern *B. antarcticus*, harbor the same numerically dominant thiotrophic Gammaproteobacteria. However, anlaysis of molecular variance of the variable sequences of six protein-coding genes of the endosymbionts revealed a strong genetic disconnection due to the formation of the Easter Microplate, which is also responsible for the subdivision of allopatric host species. We found no evidence for adaptive differentiation between the northern and southern symbiont groups but Isolation-by-Distance in the protein-coding genes. The age of Easter Microplate, 2.5–5.3 million years ago, enabled us to estimate synonymous substitution rates of the protein-coding genes, 0.77–1.62%/nucleotide/million years, which turned out to be remarkably similar to those of *E. coli* and endosymbionts of aphids. Finally, the unexpected high heterogeneity of symbiont sequences among host individuals sampled from the same location suggested that stochasticity associated with initial infections was amplified as symbionts proliferated within the host individuals.

## Additional files

Additional file 1: Table S1. Primer sequences for *16S* and six protein-coding genes of *Bathymodiolus* mussels’ symbionts. **Table S2.** Barcodes used for the amplification of (A) symbiont *16S* rRNA encoding gene from 45 individual host mussels and (B) symbiont protein-coding genes from 72 individuals of host mussel. **Table S3.** Bacterial symbiont community in the gill tissues of host mussels, *B. thermophilus* and *B. antarcticus*, based on high-throughput data of *16S* ribosomal RNA encoding gene from 45 individual host mussels. **Table S4.** Frequency distribution of *Bathymodiolus* symbiotic bacteria *16S* phylotypes at seven vent localities. **Table S5.** Genetic diversity indices of symbiont *16S* rRNA encoding gene from 45 individuals of host *Bathymodiolus* mussels in the EPR and PAR. **Table S6.** Summary table of synonymous and nonsynonymous substitutions of six protein-coding genes between two geographic groups, EPR + GAR and PAR. **Table S7.** Genetic diversity indices of symbiont protein-coding genes from 72 individuals of host *Bathymodiolus* mussels in the EPR, GAR, and PAR (based on 1% error correction criterion for the 454 pyrosequencing raw data). (DOCX 25 kb)

Additional file 2: Figure S1. Alignment of seven examined genes and *Bathymodiolus* thiotrophs incomplete genome. Dark-green larger rectangle represents incomplete genome of thiotrophic endosymbionts obtained from *Bathymodiolus* host individual at latitude of 9°N, East Pacific Rise. Small light-green rectangles represent seven target genes used in the present study. Arrows represent direction of genes. Numbers indicate position of genes in the incomplete genome. **Figure S2.** Schematic overview of our protocol for the correction of 454-pyrosequencing data of protein-coding genes of symbiotic bacteria. **Figure S3.** Hierarchical fixation indices of *F*_SC_, *F*_ST_, and *F*_CT_ calculated from each protein-coding gene across various error-correction criteria (1to 10%) applied to the raw high-throughput sequence reads. The setting of geographical groups into two regions (EPR + GAR and PAR) is shown in Figs. 1 and 2. Colors represent different protein-coding genes. As shown with the aid of lines, almost all the *F* statistics are stable irrespective of the different error-correction criteria. **Figure S4.** Mussel symbiont protein-coding gene trees from two representative sequences obtained from the EPR + GAR and PAR in this study and from other symbiont specific sequences [41]. Black squares (■) are the representative sequences of symbionts from this study. Colored boxes identify symbionts based upon ocean residence: West Pacific (orange), East Pacific (green), and Indian (blue). (DOCX 636 kb)

Additional file 3: Developed genotyping script for correcting errors in protein-coding sequences. (DOCX 18 kb)

Additional file 4: Table S3. Bacterial symbiont community in the gill tissues of host mussels,* B. thermophilus* and *B. antarcticus*, based on NGS data of *16S* ribosomal RNA gene from 45 individual host mussels. (XLSX 16 kb)

Additional file 5: Table S5. Genetic diversity indices of symbiont *16S* rRNA gene from 45 individuals of host *Bathymodiolus* mussels in the EPR and PAR. (XLSX 13 kb)

Additional file 6: Table S7. Genetic diversity indices of symbiont protein-coding genes from 72 individuals of host *Bathymodiolus* mussels in the EPR, GAR, and PAR (based on 1% error correction criterion for the 454 pryosequencing raw data). (XLSX 48 kb)

## Abbreviations

*16S* rRNA: *16S* ribosomal RNA
AMOVA: Analysis of molecular variance
*COI*: Cytochrome C oxidase subunit I
*dnaK*: Chaperone protein
EPR: East Pacific Rise
GAR: Galápagos Rift
HOV: Human occupied vehicle
IBD: Isolation-By-Distance
MID: Multiplex Identifier sequences
MY: million years
PAR: Pacific-Antarctic Ridge
*pgi*: Glucose-6-phosphate isomerase
*pykF*: Pyruvate kinase
*rpoD*: RNA polymerase, sigma D factor
SEPR: southern East Pacific Rise
*soxA*: Sulfur oxidation protein A
