# Supplementary material for: Geographical structure of endosymbiotic bacteria hosted by Bathymodiolus mussels at eastern Pacific hydrothermal vents
Source: BMC Evol Biol. 2017 May 30;17:121. doi: 10.1186/s12862-017-0966-3 (PMC5450337; doi:10.1186/s12862-017-0966-3)
Supplement: Supplementary file 1 — Primer sequences for 16S and six protein-coding genes of Bathymodiolus mussels’ symbionts. Table S2. Barcodes used for the amplification of (A) symbiont 16S rRNA encoding gene from 45 individual host mussels and (B) symbiont protein-coding genes from 72 individuals of host mussel. Table S3. Bacterial symbiont community in the gill tissues of host mussels, B. thermophilus and B. antarcticus, based on high-throughput data of 16S ribosomal RNA encoding gene from 45 individual host mussels. Table S4. Frequency distribution of Bathymodiolus symbiotic bacteria 16S phylotypes at seven vent localities. Table S5. Genetic diversity indices of symbiont 16S rRNA encoding gene from 45 individuals of host Bathymodiolus mussels in the EPR and PAR. Table S6. Summary table of synonymous and nonsynonymous substitutions of six protein-coding genes between two geographic groups, EPR + GAR and PAR. Table S7. Genetic diversity indices of symbiont protein-coding genes from 72 individuals of host Bathymodiolus mussels in the EPR, GAR, and PAR (based on 1% error correction criterion for the 454 pyrosequencing raw data). (DOCX 25 kb) [file 12862_2017_966_MOESM1_ESM.docx]

Research article

Geographical structure of endosymbiotic bacteria hosted by *Bathymodiolus* mussels at eastern Pacific hydrothermal vents

Phuong-Thao Ho^1^, Eunji Park^2^, Soon Gyu Hong^3^, Eun-Hye Kim^3^, Kangchon Kim^1^, Sook-Jin Jang^1^, Robert C. Vrijenhoek^4^, and Yong-Jin Won^1,2†^

# Additional file 2

Table S1: Primer sequences for *16S* and six protein-coding genes of *Bathymodiolus* mussels’ symbionts

| Genetic marker |  | 5’ 🡪 3’ | Expected size (bp) | Nested PCR |
| --- | --- | --- | --- | --- |
| *16S* | F^a^ | barcode^b^ – linker(AC) – AGAGTTTGATCMTGGCTCAG | 490 | 1st PCR |
|  | R | barcode – linker(AC) – GWATTACCGCGGCKGCTG |  |  |
| Cytochrome c oxidase subunit I  (*COI*) | F | CATCACGGCCCTGAAAAAGG | 760 | 1st PCR |
|  | R | ATAATATACACCTCAGGATG |  |  |
|  | F | barcode^c^ – ACTGACTACGATGCACGGCT | 480 | 2st PCR |
|  | R | barcode – AATGCGGTACCAAAGTTACG |  |  |
| Chaperone protein  (*dnaK*) | F | ATTATCGGTATTGACTTAGG | 800 | 1st PCR |
|  | R | TTTAAGCGTTGTAGTGCCAT |  |  |
|  | F | Barcode – AGGTGATCGCACCACACCCT | 510 | 2st PCR |
|  | R | barcode – AGGTACCGCCACCTAAGTCA |  |  |
| Glucose-6-phosphate isomerase  (*pgi*) | F | TTTGCCTTATCAAGATACCA | 600 | 1st PCR |
|  | R | AGAGAAAATCGCATTAATAT |  |  |
|  | F | barcode – AATTAAGGCGTATGTTGAGA | 480 | 2st PCR |
|  | R | barcode – ACTATCCGACACACGCCTAG |  |  |
| Pyruvate kinase  (*pykF*) | F | TTAGATGGCACCGATGCAGT | 500 | 1st PCR |
|  | R | TTCATCAGATTAGTGCCACC |  |  |
|  | F | barcode – TGCTGAAACAGCAGCAGGCG | 450 | 2st PCR |
|  | R | barcode – ACATGCATTCCTTTGGTTAA |  |  |
| RNA polymerase, sigma D factor  (*rpoD*) | F | ATTGAAACCATTAACAAACT | 450 | 1st PCR |
|  | R | CTCTAGGAAACTTTGCAATT |  |  |
|  | F | barcode – AATCGTGTTAAACGCCAGTT | 410 | 2st PCR |
|  | R | barcode – TGCGCCCTAGAAGGGTG |  |  |
| Sulfur oxidation protein A  (*soxA*) | F | ATGAGGAAAATTATAACAAC | 493 | 1st PCR |
|  | R | AGAAATCTCCATGCCATTAC |  |  |
|  | F | barcode – AATGACTTTTCCAAAGGTCC | 400 | 2st PCR |
|  | R | barcode – TTGCCTGCGTTATAAGCAGC |  |  |

^a^ F: Forward, R: Reverse

^b^ Barcodes comprising eight nucleotides were used for the amplicons of symbiont *16S* rRNA encoding gene for forty-five individual host mussels (Supplementary Table S3A).

^c^ Barcodes comprising eight nucleotides were used for the amplicons of symbiont protein-coding genes for seventy-two individual host mussels (Supplementary Table S3B).

Table S2: Barcodes used for the amplification of (A) symbiont *16S* rRNA encoding gene from forty-five individual host mussels and (B) symbiont protein-coding genes from seventy-two individuals of host mussel

(A)

| Sample | Barcode for individual | Sample | Barcode for individual |
| --- | --- | --- | --- |
| A^a^2498 #^b^2 | ACACACTG | A3339 #3 | CAGTGAGA |
| A2498 #3 | ACAGACAG | A4091 #92 | CATGAGCT |
| A2498 #12 | ACAGAGAC | A4091 #94 | CGATCGAT |
| A3320 #6 | ACAGCAGA | A4091 #96 | CGCGATAT |
| A3320 #7 | ACAGTCAC | A4091 #98 | CGTAGCTA |
| A3320 #8 | ACGACATC | A3540 #73 | CTGACTCT |
| A3323 #16 | AGACACTC | A3540 #87 | CTGAGTGT |
| A3323 #17 | AGACTCAC | A3540 #85 | CTGATCTC |
| A3323 #18 | AGAGTGTG | A3540 #72 | CTGTACTC |
| A3323 #19 | AGCAGATG | A3540 #81 | CTGTCACT |
| A3323 #20 | AGCTAGCT | A3540 #78 | CTGTGTCT |
| A3323 #21 | AGTCACAC | A3540 #74 | GACACTGT |
| A3323 #22 | AGTCAGAG | A3540 #70 | GACAGTCT |
| A3323 #24 | AGTCGTGT | A3540 #95 | GACATCAC |
| A3327 #1 | AGTGAGAC | A3340 #27 | GACTAGTC |
| A3327 #9 | AGTGTCAC | A3340 #14 | GAGACACA |
| A3327 #5 | ATATCGCG | A3340 #1 | GAGAGTGT |
| A3327 #10 | ATCGTAGC | A3340 #24 | GAGTACTC |
| A3327 #14 | ATGCTAGC | A3340 #7 | GAGTCTGT |
| A3327 #19 | CACACAGT | A3340 #16 | GATCGTAC |
| A3327 #20 | CACAGAGA | A3340 #5 | GATGCATC |
| A4096 #1 | CACATCTC | A3340 #10 | GCATATCG |
| A4096 #2 | CACTACTC | - | - |

^a^ DSV *Alvin*

^b^ Serial number of sampling individuals

(B)

| Serial no. of individuals | Barcode for each individual |
| --- | --- |
| 1 | ATGC |
| 2 | AGCT |
| 3 | ACGT |
| 4 | TAGC |
| 5 | TGAT |
| 6 | TCTG |
| 7 | CTGT |
| 8 | CATA |

Table S3. Bacterial symbiont community in the gill tissues of host mussels, *B. thermophilus* and *B. antarcticus*, based on high-throughput data of *16S* ribosomal RNA encoding gene from forty-five individual host mussels

Table S4: Frequency distribution of *Bathymodiolus* symbiotic bacteria *16S* phylotypes at seven vent localities

| Phylotype | 9°N | 7°S | 11°S | 17°S | 23°S | 32°S | 38°S |
| --- | --- | --- | --- | --- | --- | --- | --- |
| HT1 | 13110 | 1836 | 7456 | 6592 | 1874 | 6371 | 3001 |
| HT2 | 38 | 167 | 5 | 0 | 0 | 0 | 0 |
| HT3 | 28 | 0 | 21 | 25 | 19 | 13 | 6 |
| HT4 | 42 | 5 | 0 | 12 | 0 | 11 | 11 |
| HT5 | 36 | 0 | 16 | 24 | 0 | 0 | 5 |
| HT6 | 33 | 0 | 7 | 20 | 6 | 0 | 23 |
| HT7 | 23 | 0 | 26 | 15 | 0 | 5 | 0 |

Table S5. Genetic diversity indices of symbiont *16S* rRNA encoding gene from 45 individuals of host *Bathymodiolus* mussels in the EPR and PAR

Table S6: Summary table of synonymous and nonsynonymous substitutions of six protein-coding genes between two geographic groups, EPR+GAR and PAR

| Type of change | Gene | Fixed differences | Polymorphisms |
| --- | --- | --- | --- |
| Synonymous | *COI* | 5 | 18 |
|  | *dnaK* | 8 | 26 |
|  | *pgi* | 5 | 33 |
|  | *pykF* | 2 | 32 |
|  | *rpoD* | 1 | 29 |
|  | *soxA* | 4 | 15 |
| Nonsynonymous | *COI* | 0 | 1 |
|  | *dnaK* | 0 | 4 |
|  | *pgi* | 1 | 6 |
|  | *pykF* | 0 | 5 |
|  | *rpoD* | 0 | 2 |
|  | *soxA* | 0 | 10 |

Table S7. Genetic diversity indices of symbiont protein-coding genes from 72 individuals of host *Bathymodiolus* mussels in the EPR, GAR, and PAR (based on 1% error correction criterion for the 454 pyrosequencing raw data)
