# Supplementary material for: Geographical structure of endosymbiotic bacteria hosted by Bathymodiolus mussels at eastern Pacific hydrothermal vents
Source: BMC Evol Biol. 2017 May 30;17:121. doi: 10.1186/s12862-017-0966-3 (PMC5450337; doi:10.1186/s12862-017-0966-3)
Supplement: Supplementary file 2 — Alignment of seven examined genes and Bathymodiolus thiotrophs incomplete genome. Dark-green larger rectangle represents incomplete genome of thiotrophic endosymbionts obtained from Bathymodiolus host individual at latitude of 9°N, East Pacific Rise. Small light-green rectangles represent seven target genes used in the present study. Arrows represent direction of genes. Numbers indicate position of genes in the incomplete genome. Figure S2. Schematic overview of our protocol for the correction of 454-pyrosequencing data of protein-coding genes of symbiotic bacteria. Figure S3. Hierarchical fixation indices of F SC, F ST, and F CT calculated from each protein-coding gene across various error-correction criteria (1to 10%) applied to the raw high-throughput sequence reads. The setting of geographical groups into two regions (EPR + GAR and PAR) is shown in Figs. 1 and 2. Colors represent different protein-coding genes. As shown with the aid of lines, almost all the F statistics are stable irrespective of the different error-correction criteria. Figure S4. Mussel symbiont protein-coding gene trees from two representative sequences obtained from the EPR + GAR and PAR in this study and from other symbiont specific sequences [41]. Black squares (■) are the representative sequences of symbionts from this study. Colored boxes identify symbionts based upon ocean residence: West Pacific (orange), East Pacific (green), and Indian (blue). (DOCX 636 kb) [file 12862_2017_966_MOESM2_ESM.docx]

Research article

Geographical structure of endosymbiotic bacteria hosted by *Bathymodiolus* mussels at eastern Pacific hydrothermal vents

Phuong-Thao Ho^1^, Eunji Park^2^, Soon Gyu Hong^3^, Eun-Hye Kim^3^, Kangchon Kim^1^, Sook-Jin Jang^1^, Robert C. Vrijenhoek^4^, and Yong-Jin Won^1,2†^

# Additional file 1


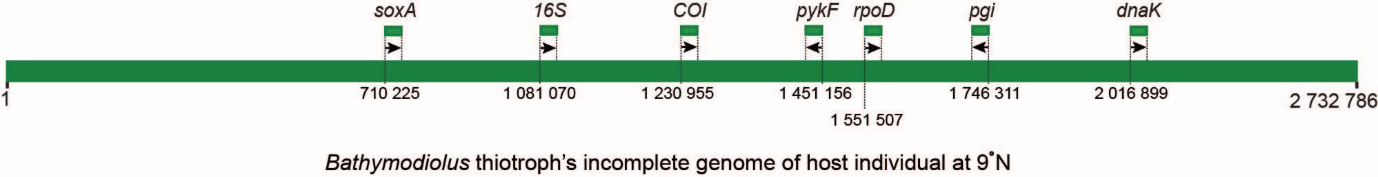


Figure S1: Alignment of seven examined genes and *Bathymodiolus* thiotrophs incomplete genome. Dark-green larger rectangle represents incomplete genome of thiotrophic endosymbionts obtained from *Bathymodiolus* host individual at latitude of 9°N, East Pacific Rise. Small light-green rectangles represent seven target genes used in the present study. Arrows represent direction of genes. Numbers indicate position of genes in the incomplete genome.


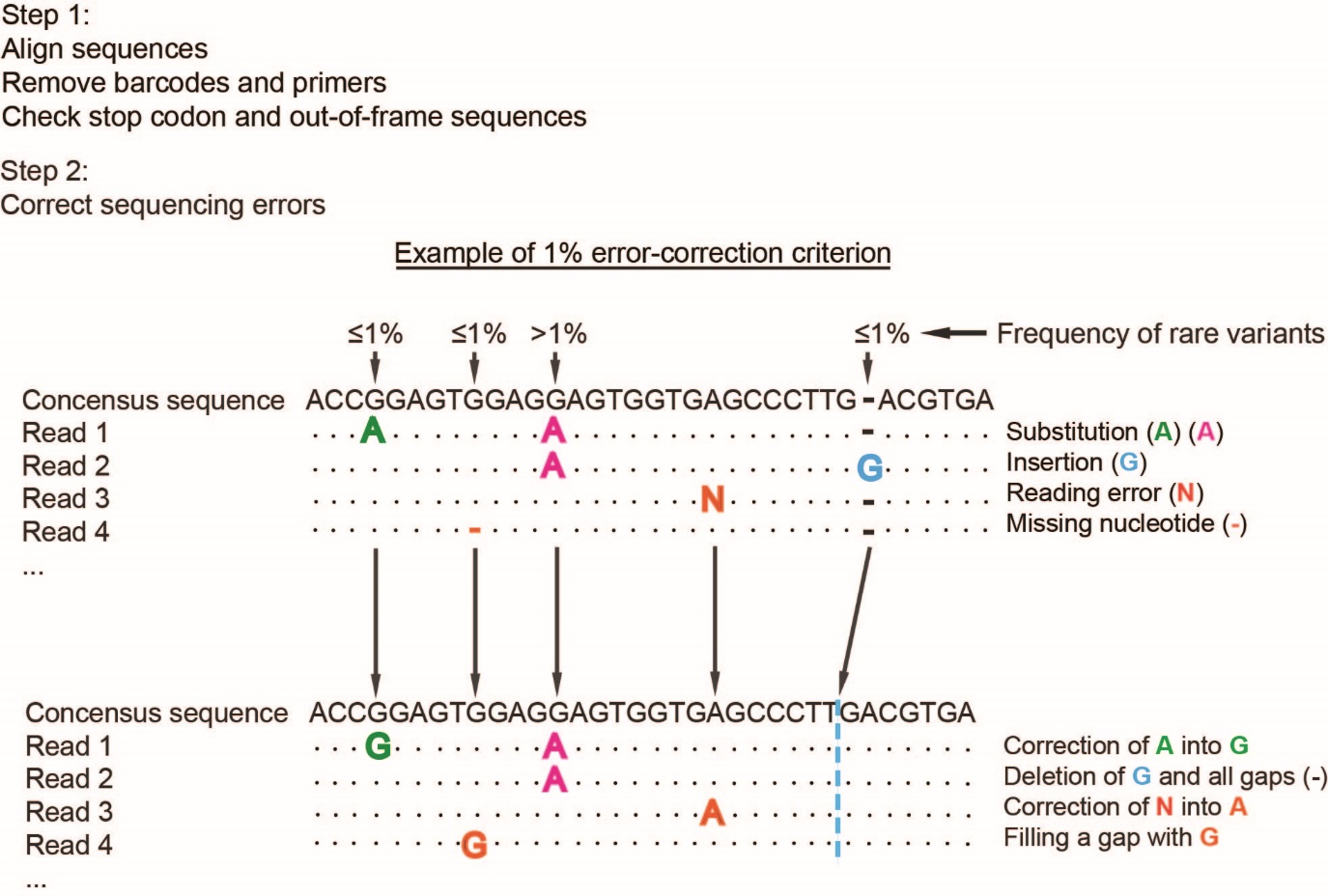


Figure S2: Schematic overview of our protocol for the correction of 454-pyrosequencing data of protein-coding genes of symbiotic bacteria. Here, we illustrate our logic for detecting some sequencing errors based on the rarity of them compared to real nucleotides at those homologous sites. First, we corrected ambiguous nucleotides (N) and fill all missing nucleotides (gaps) with corresponding nucleotide in the consensus sequence. In principle, we assumed that erroneous nucleotide states of sequence reads in a certain DNA site probably have very low frequency than real states at that site: for example, less than or equal to 1% of total reads as illustrated in this figure. We utilized this kind of arbitrary but low frequency of rare variants for defining errors and correcting them into a most common type of nucleotide at that site, and called this arbitrary criterion as ‘1% error-correction criterion’. As illustrated in this figure, if the frequency of rare variants at one site is less than or equal to 1%, the rare variants are considered as sequencing errors and will be corrected into the most common state of nucleotide at that site. If the frequency of rare variants is more than 1%, those variants are accepted as real nucleotides, and thus the rare variants will not be corrected.


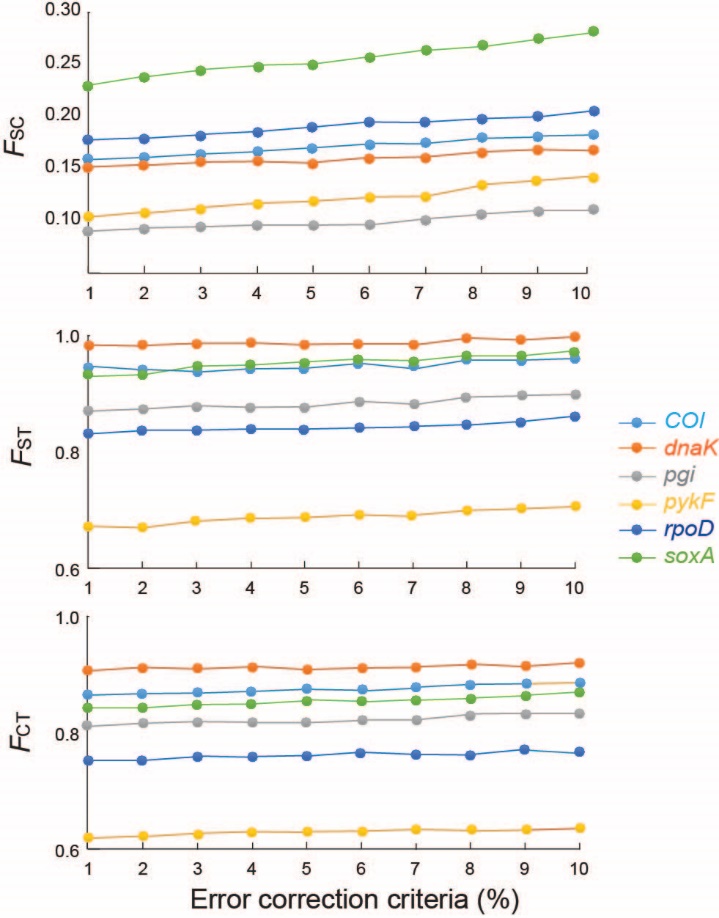


Figure S3: Hierarchical fixation indices of *F*_SC_, *F*_ST_, and *F*_CT_ calculated from each protein-coding gene across various error-correction criteria (1to 10%) applied to the raw high-throughput sequence reads. The setting of geographical groups into two regions (EPR and PAR) is shown in Figures 1 and 2. Colors represent different protein-coding genes. As shown with the aid of lines, almost all the *F* statistics are stable irrespective of the different error-correction criteria.


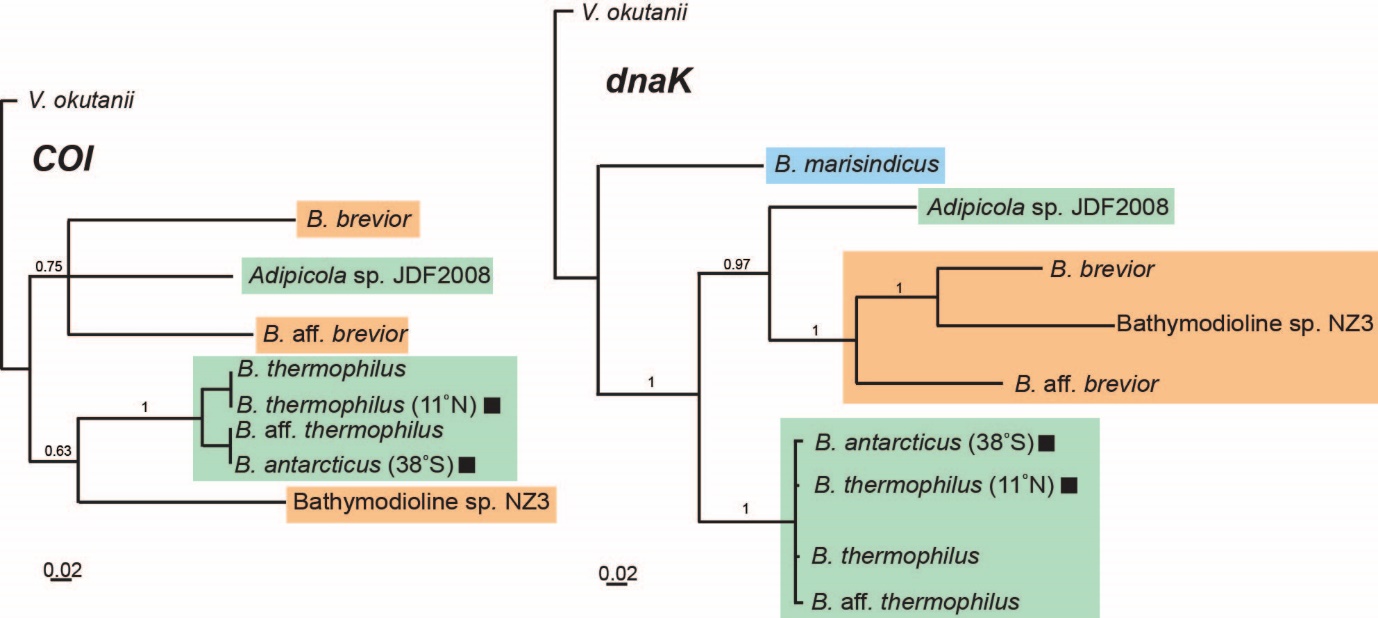


Figure S4: Mussel symbiont protein-coding gene trees from two representative sequences obtained from the EPR and PAR in this study and from other symbiont specific sequences [41]. Black squares (■) are the representative sequences of symbionts from this study. Colored boxes identify symbionts based upon ocean residence: West Pacific (orange), East Pacific (green), and Indian (blue).
