# Supplementary material for: Geographical structure of endosymbiotic bacteria hosted by Bathymodiolus mussels at eastern Pacific hydrothermal vents
Source: BMC Evol Biol. 2017 May 30;17:121. doi: 10.1186/s12862-017-0966-3 (PMC5450337; doi:10.1186/s12862-017-0966-3)
Supplement: Supplementary file 3 — Developed genotyping script for correcting errors in protein-coding sequences. (DOCX 18 kb) [file 12862_2017_966_MOESM3_ESM.docx]

Research article

Geographical structure of endosymbiotic bacteria hosted by *Bathymodiolus* mussels at eastern Pacific hydrothermal vents

Phuong-Thao Ho^1^, Eunji Park^2^, Soon Gyu Hong^3^, Eun-Hye Kim^3^, Kangchon Kim^1^, Sook-Jin Jang^1^, Robert C. Vrijenhoek^4^, and Yong-Jin Won^1,2†^

# Additional file 6

Developed genotyping script for correcting errors in protein-coding sequences:

def count_numbases(sequence, loc):

numA = 0

numC = 0

numG = 0

numT = 0

numH = 0

numN = 0 #count the number of ambiguous bases

for seq in sequence:

if seq[loc] == 'A': numA += 1

elif seq[loc] == 'C': numC += 1

elif seq[loc] == 'G': numG += 1

elif seq[loc] == 'T': numT += 1

elif seq[loc] == '-': numH += 1

else: numN += 1

base_count = [numA, numC, numG, numT, numN, numH]

return base_count

e_threshold = 1

from os import listdir

from os.path import isfile, join

inpath = './functional_genes_input/'

outpath = './functiona_genes_output/'

files = [ f for f in listdir(inpath) if isfile(join(inpath,f)) ]

for file in files:

infname = inpath+file

filename = file.replace(" ", "_")

outfname = outpath+filename.split(".")[0] + '_ec_' + str(e_threshold) + "." + file.split(".")[1]

inf = open(infname, "r")

outf = open(outfname, "w")

#Put the data in lists

info = []

sequence = []

for line in inf:

if line.find(">") >= 0:

info.append(line)

else:

seq = []

for base in line:

seq.append(base)

sequence.append(seq)

#Check error-like locus and correct

bases = ['A', 'C', 'G', 'T', 'N', '-']

for loc in range(0, len(sequence[0])):#The sequences in a file should be aligned and have equal length

if sequence[0][loc] == '\n' or sequence[0][loc] == '\r':

break

base_count = count_numbases(sequence, loc)

#1. Indel or shifted locus

if base_count[5] > 0:

if base_count[5] <= 0.5*len(sequence): #"-"s is to be replaced with the consensus among none-"-"

consensus = bases[base_count.index(max(base_count[0:5]))]

for seq in sequence:

if seq[loc] == '-':

seq[loc] = consensus

base_count = count_numbases(sequence, loc)

elif base_count[5] > 0.5*len(sequence): #This locus is to be removed

for seq in sequence:

seq[loc] = '-'

continue #to next locus

#2. Low frequency mutations replaced by the most frequent allele, depending on the frequency of the allele

print loc, 1.0*max(base_count)/len(sequence), 0.01*(100 - e_threshold)

A = (1.0*max(base_count)/len(sequence))

B = (0.01*(100 - e_threshold))

if A > B or A==B:

print loc

consensus = bases[base_count.index(max(base_count[0:5]))]

for seq in sequence:

seq[loc] = consensus

for i in range(0, len(sequence)):

outf.write(info[i])

seq = ''

for s in sequence[i]:

if s != '-':

seq += s

outf.write(seq)

inf.close()

outf.close()
